# Chronic Pulmonary Histoplasmosis—A Scoping Literature Review

**DOI:** 10.1093/ofid/ofaa119

**Published:** 2020-04-06

**Authors:** Jacob Baker, Chris Kosmidis, Anna Rozaliyani, Retno Wahyuningsih, David W Denning

**Affiliations:** 1 The University of Manchester and the Manchester Academic Health Service Centre, Manchester, UK; 2 The National Aspergillosis Centre, Wythenshawe Hospital, Manchester University NHS Foundation Trust, Manchester, UK; 3 Universitas Indonesia, Faculty of Medicine, Jakarta, Indonesia; 4 Universitas Kristen Indonesia, Faculty of Medicine, Jakarta, Indonesia

**Keywords:** cavitation, chronic pulmonary histoplasmosis, *Histoplasma*, nodule, tuberculosis

## Abstract

Chronic pulmonary histoplasmosis (CPH) is an uncommon manifestation of *Histoplasma* infection with features similar to pulmonary tuberculosis (TB). In endemic areas, it may be misdiagnosed as smear-negative pulmonary TB. Historical case series mainly from patients with presumed TB described a high frequency of cavitation and poor prognosis, likely resulting from delayed presentation. More recent reports suggest that CPH can present with nodules, lymphadenopathy, or infiltrates, with cavities being a less common feature. Emphysema is the main risk factor for cavitary CPH. CPH is therefore an umbrella term, with chronic cavitary pulmonary histoplasmosis and *Histoplasma* nodules being the main long-term manifestations in nonimmunocompromised individuals. Diagnosis relies on a high index of suspicion, use of fungal culture of respiratory samples, antibody testing, and compatible radiological picture. Treatment with itraconazole for at least 12 months is recommended. Morbidity from CPH results from slow progression of cavities and gradual loss of lung function, especially if not recognized and treated. Studies on the epidemiology of CPH are needed in order to improve understanding of the disease.

*Histoplasma capsulatum* var. *capsulatum* (Hcc) is a dimorphic fungus [1] transmitted via the airborne route, particularly during activities like caving, construction work, and excavations [2]. It is endemic to the Ohio River and Mississippi River valleys [3], but more recent investigation has shown a large number of cases in previously nonendemic areas of the United States [4] and Eastern Canada [5, 6]. In addition, recent articles have collated older studies to show the distribution of Hcc in South Africa [7], India [8], Oceania [9], China [10], Africa [11], Central and South America and Europe [12], and Southeast Asia [13].

Histoplasmosis can present in several forms. The disseminated form affects immunocompromised patients, carries a high morbidity and mortality, and has been a significant problem during the HIV epidemic [14]. The acute pulmonary form (acute pulmonary histoplasmosis) is a mild disease in people without prior immune compromise.

Chronic cavitary pulmonary histoplasmosis (CCPH) can develop after acute pulmonary histoplasmosis [15] and is marked by low-grade chronic symptoms, persistent cavitation, and development of pulmonary fibrosis and progressive pulmonary insufficiency [16, 17]. The predisposition to cavitation in the upper lobes, particularly the apical and apical-posterior segments, is similar to pulmonary tuberculosis (TB) and chronic pulmonary aspergillosis (CPA) [16]. More recent series have emphasized that nodules are a more common manifestation of chronic histoplasma infection, so in this review we use the term CCPH to refer to those with cavitary disease and the term chronic pulmonary histoplasmosis (CPH) for all forms of chronic lung infection caused by *Histoplasma*, including *Histoplasma* nodules.

Because of its clinical presentation, CCPH can cause considerable diagnostic confusion with TB. Although another chronic fungal infection, chronic pilmonary aspergillosis or CPA, has recently attracted attention as a differential diagnosis of TB in endemic areas [18]. There is surprisingly little data available on the global burden of CPH. The various features and forms of CPH are discussed in more detail below with a focus on the changing understanding of the disease over the years.

## METHODS

The collection strategy for the literature review for CPH was focused on key large case series [15, 16, 19]. Additional series were found through citations in these publications [20, 21]. Additional publications were found using PubMed and Google Scholar.

Reviewing clinical literature to describe CPH presents 2 problems. First, in more recently gathered clinical data, the focus has tended to be on disseminated rather than pulmonary disease. Some studies describe the clinical features of both disseminated and pulmonary disease without distinction, making it difficult to describe CPH alone. The second issue concerns the change in the description of the disease entity of CPH. Historic studies tended to categorize it by radiographic presence of cavitation or other features, whereas modern studies categorize it by the chronicity of symptoms. Here the term CPH is used to describe all chronic forms of pulmonary histoplasmosis, with cavitary disease being a subsection.

### Relationship Between CCPH and Tuberculosis

The clinical similarity to reactivation pulmonary TB remains the principal challenge in identifying and managing CCPH. It is likely that some smear (and culture or GeneXpert)-negative cases of pulmonary tuberculosis in areas endemic of histoplasmosis are misdiagnosed cases of CCPH. In the United States in the 1950s, when tuberculosis was more prevalent, one study found that 7.2% of patients in a Missouri TB sanatorium had CCPH [22]. Many other case series have included such misdiagnosed cases [16, 22, 23]. Typically, CCPH was only considered after months of ineffective antimycobacterial therapy [24]. High MDR-TB and histoplasmosis burden may coexist [25] in areas such as India [8], China [10], and Africa [11]. Considering the reduced technical diagnostic facilities and health care funding available in some areas, this increases the chances of misdiagnosis. Unlike the connection between TB and subsequent development of CPA, there is no evidence that TB predisposes to subsequent development of CPH. TB and CPH can occur simultaneously [16]. CPH has some similarities to other chronic pulmonary fungal infections [26].

### Etiology

The risk factors for the development of CCPH were described by Goodwin et al. [16] and Wheat et al. [15]. However, their distinct approaches created different results. Goodwin et al. included patients who had largely been misdiagnosed as TB previously, creating a study group with more advanced disease. It was therefore difficult to make any judgment on what made these individuals develop cavitary disease without a control group. A study based on patients previously thought to have TB had a cavitation rate of 87% [20]. These studies overestimated the rates of cavitation in CCPH [19].

Conversely, Wheat et al. studied a cohort with acute pulmonary histoplasmosis during 2 outbreaks and compared those who developed cavitary disease (8% of all cases) with those who did not [15]. Factors significantly associated with cavitation were older age, male sex, white race, preexisting immunosuppression and, most strongly, preexisting chronic lung disease. A similar study showed a lower rate (1.8%) of progression to cavitation following infection [27].

More recent case series consider CPH as an entity defined by the chronicity of symptoms rather than the development of cavitation [19]. A collation of studies found a cavitation rate ranging from 33% to 66% [28]. A retrospective review of CPH cases based on chronicity of symptoms rather than presence of cavitation identified a different set of risk factors for chronic disease with cavitation, cavitation being noted in 39% of these patients (Table 1). Smoking and the presence of chronic lung disease continue to be shown as potential risk factors for CPH but show a greater association with the development of cavities. CPH tends to affect older people [19], likely due to the connection between age and emphysema [24].

**Table 1.** Characteristics of 46 CPH Patients, Median Patient Age (Range) 56 (20–85) Years [19]

Clinical Factors	All CPH, %	CCPH, %	*Histoplasma* Nodules, %	*P* Value (Compared With Cavitation)
Smokers—current or former	71	89	36	.05
Male	52	72	39	.04
Positive cultures	34	50	4	.006
COPD	20	39	7	.02

Abbreviations: CCPH, chronic cavitary pulmonary histoplasmosis; COPD, chronic obstructive pulmonary disease; CPH, chronic pulmonary histoplasmosis.

### Symptomatology

CCPH was first described in 1948 and acknowledged as a separate clinical entity in 1953 [29]. The most significant symptoms were chronic cough without hemoptysis and considerable weight loss [16], with Rubin et al. also describing fever and dyspnea in the majority [20]. These symptoms were also predominant in the study of CCPH following histoplasmosis outbreaks [15]. Symptoms vary according to stage of disease (late disease being characterized by cavity development) (Table 2). Modern case series are of little value to understanding CPH as they describe the symptomatology primarily of disseminated disease [10].

**Table 2.** Signs and Symptoms in 228 Cases of Chronic Cavitary Pulmonary Histoplasmosis [16]

Signs and Symptoms	All Cases, %	Early Cases, %	Late Cases, %
Cough	61	48	76
Sputum production	42	31	61
Chest pain	25	35	20
Dyspnea	21	15	26
Hemoptysis	22	4	36
Malaise	31	34	30
Fatigability	33	30	38
Weakness	35	35	35
Feverishness	25	39	19
Night sweats	17	23	15
Chilliness	9	17	4
Anorexia or nausea	5	10	2
Fever	31	42	26
Weight loss	60	58	64

Hemoptysis is not a predominant feature in CPH; Goodwin reported it in 11% of cases [16], and if large volume, it can suggest the presence of an aspergilloma [19]. A summary of the symptoms reported in 5 studies of CPH (n = 401) is shown in Table 3.

**Table 3.** Clinical Features of 401 Cases of Chronic Pulmonary Histoplasmosis From 5 Separate Studies [16, 19, 20, 23, 29]

Clinical Feature	%
Cough	70
Chest pain	31
Hemoptysis	25
Fatigue	30
Fever	43
Weight loss	61

Both pulmonary and constitutional symptoms of CCPH are identical to those of pulmonary TB, although usually less severe. Chest pain tended to be a deep aching pain, as opposed to the pleuritic chest pain found in pulmonary TB [16]. Chronic pulmonary aspergillosis is another common differential diagnosis.

### Radiological Features

**Figure 1.**
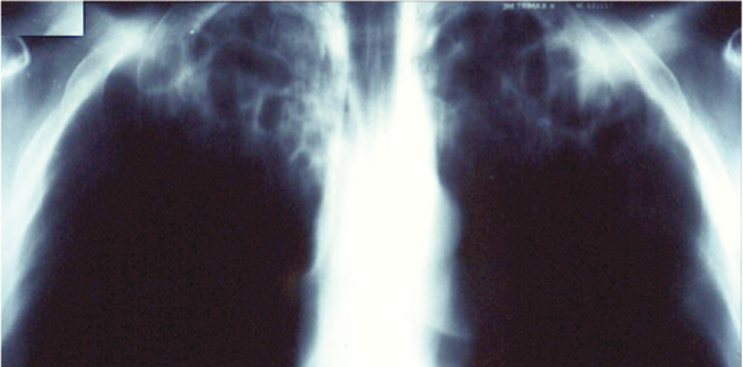
A male truck driver from Argentina who drank and smoke excessively presented with cough and hoarseness. A laryngeal biopsy showed granulomas. He was treated for tuberculosis for 6 months without improvement. A second laryngeal biopsy also showed granulomas, and he was treated for tuberculosis again. He deteriorated and required a tracheostomy. Antibody against *Histoplasma capsulatum* was detected in serum, and culture grew this organism. He responded well to itraconazole. Source: Image from Dr. Iris Nora Tiraboschi, Hospital de Clínicas, Universidad de Buenos Aires.

In the case series by Wheat et al., all but 1 of 45 (98%) patients had upper lobe cavitation, more often on the right apex (84% vs 53%) [15]. The cavities were often thick-walled (66.7%) with apical pleural thickening (44.4%) (Figure 1). Although pulmonary calcified granulomas were common (77.8%), hilar lymphadenopathy was rare (2.2%), in contrast to acute pulmonary disease. Goodwin et al. studied sequential chest x-rays to define the early features of CCPH. The earliest feature was a dense, sharply demarcated area of consolidation that was pitted with radio-translucent pockets, giving a “moth eaten or Swiss cheese”–like appearance. Over the ensuing weeks or months, the area contracts, becoming denser, until it resolves, leaving behind a cavity that can persist for months or years [23]. These cavities can progress in size to involve the entire lung lobe, the so-called “marching cavity,” a characteristic feature of CCPH where the apical cavity can slowly expand to destroy the entire lung [16, 17]. Bronchopleural fistula can also develop [15]. Computed tomography findings of CCPH are barely described in the literature.

Because of shifting understanding of CPH, older case series confirmed pathologically had a very high cavitation rate, whereas more modern studies based on the chronicity of symptoms have found a lower cavitation rate, around 30% [19]. In fact, in Kennedy and Limper, nodules were the most common feature of CPH, seen in 93% of patients on CT scans [19]. These nodules can mimic pulmonary malignancy both clinically and radiographically and can complicate cancer screening programs; in an endemic area, 61% of participants enrolled in a lung cancer screening program using low-dose CT scans had positive findings, compared with around 20% in other areas. Only 6/80 patients had cancer diagnosed [30]. In a series of 27 patients who presented with lesions suspicious of cancer eventually diagnosed as fungal disease in Brazil, histoplasmosis was the most common diagnosis (25.9%) [31].

Granulomatous inflammation in parabronchial lymph nodes caused by histoplasmosis has been described to be a common cause of calcified broncholiths, which may arise in a similar manner to TB [32].

### Laboratory Diagnosis

#### Microscopy

**Figure 2.**
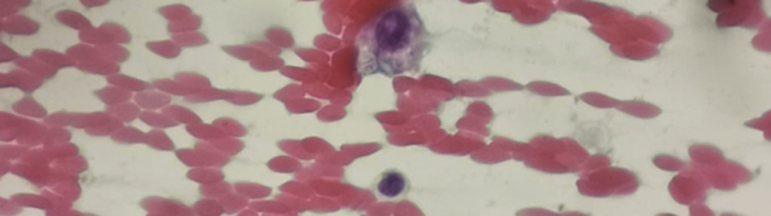
Characteristic appearances of intracellular *Histoplasma capsulatum* var. *capsulatum* organisms from a percutaneous biopsy. Source: Image from Dr. Anna Rozaliyani, Universitas Indonesia, Jakarta.

Microscopy of sputum for Hcc is not a useful tool for the routine diagnosis of CCPH. Although silver stain of tissue sections (Figure 2) or Wright stain of peripheral blood smears can be a rapid, but insensitive, diagnostic tool in disseminated disease [21], its low sensitivity and specificity make it ineffective in CCPH. Unlike *Candida* species, *Histoplasma* is rarely seen on gram stain.

#### Culture

Culture remains the gold standard of diagnosis but may take up to 8 weeks and sensitivity is poor. Goodwin et al. found that only one-third of 2000 sputum samples from CCPH patients cultured *Histoplasma,* rising to 44% in cavitary disease [16]. Positive sputum culture was seen in 50% of postoutbreak cases [15]. Sputum induction or more invasive techniques may be required to obtain specimens [21]. In another study, 46% of 902 sputum samples and 39% of 44 gastric washings were positive. Bronchial washings delivered positive cultures in 6 of 9 (66.7%) cases [20] and 6 of 26 (23%) washes [19].

#### Skin Testing

The histoplasmin sensitivity skin test can provide a clue to the diagnosis, but must be combined with other tests and is less useful in areas endemic to histoplasmosis due to significant background exposure to Hcc [33]. Histoplasmin is not commercially available currently, so this is not currently realistic in clinical practice.

#### Histoplasma Antibody

The chronic nature of CCPH and the usual immune competence of the patients make CCPH suitable for diagnosis by antibody detection [17]. Antibody can be detected through the immunodiffusion (ID) test and complement fixation (CF). The ID test identiﬁes H and M precipitin bands to Hcc antigen. It is slightly more specific but less sensitive than CF, so it should not be used for screening [33]. CF antibodies remain for years after infection, so a single low CF titer means only that the patient was previously exposed to Hcc [16]. Although 95% of patients with histoplasmosis show raised titers, around a quarter of cases will have lower titers than the standard diagnostic level at 1:8 or 1:16. Although this could simply be previous exposure, lower titers should not be discounted, as some of them may have active disease [33]. A recently published series of six patients with CPH found 5 (83.3%) to have detectable antibody [34]. There is a degree of cross-reaction of the CF assay with other fungal infections. A study of 228 cases of culture-proven CCPH found the CF assay to be positive in 74%; there was no correlation between CF titer and outcome [16]. An additional complication of serological diagnosis is a base level of positivity in areas of endemicity: 0.5% in ID and 4% in CF [15]. Furthermore, the commercially available tests in the United States varied in their diagnostic performance in the 1980s [34]. A Western blot assay showed high performance characteristics in a recent large series from Brazil but is not commercialized [35].

Similar to CPA, antibody detection may help in differentiating CPH from TB and has been broadly established for the diagnosis of histoplasmosis [17]. CF testing is a slightly more sensitive (95%) but less specific test than the ID test (34% positives in patients with confirmed TB) [21]. This makes ID a better test for the differentiation of smear-negative tuberculosis and CCPH where a positive culture has not been obtained, as only 5% of confirmed TB cases had a positive *Histoplasma* antibody. In contrast to *Aspergillus* nodules, no data are available on the performance of *Histoplasma* antibody detection for *Histoplasma* nodules [36].

#### Antigen Detection

Antigen detection was first described in the urine and serum in 1986 [21]. Previously based on a solid phase radioimmunoassay, the current and more effective method is the enzyme immunoassay [33]. Antigen detection has predominantly been useful in disseminated disease [17]. It is a poor diagnostic tool in CPH, as only 10%–20% of cases produce a positive result [33]. Another study showed a much higher rate of detection in CPH (87.5%) but with a small sample size (n = 8) [37]. Bronchoalveolar lavage also provides samples for antigen testing: positive in pulmonary histoplasmosis in 70%–84% [33, 36, 38]. An additional limitation of antigen detection is significant cross-reaction with other fungal antigens [36]. The low specificity of antigen testing makes it unsuitable for differentiating histoplasmosis from other fungal infections.

Given the poor sensitivity of culture, the often low levels of *Histoplasma* antibody, the very low sensitivity of *Histoplasma* antigen in blood, and the modest sensitivity in bronchoalveolar lavage, multiple tests may be required to confirm the diagnosis. If a misdiagnosis of CPA is made instead of CPH and treated with itraconazole or another azole, CPH should resolve without the diagnosis ever being made.

### Natural History

**Figure 3.**
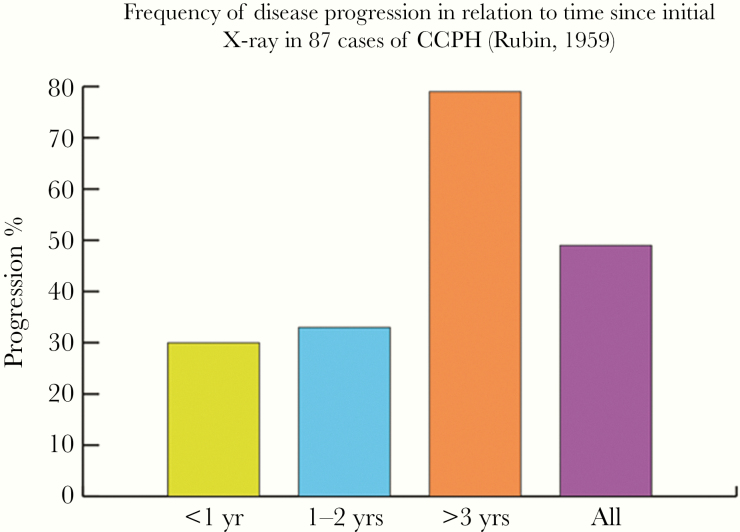
Frequency of disease progression in relation to time since initial x-ray in 87 cases of chronic cavitary pulmonary histoplasmosis [20].

The prognosis of CPH was initially believed to be rather poor, based on studies with selection bias including only patients with late-stage disease [23]. Multiple large-scale studies were performed in the United States with long-term follow-up to examine prognosis. Rubin et al. followed 90 patients with CCPH in the 1950s for an average of 40 months [20]. Nine died as a direct result of their pulmonary disease, 4 following pulmonary surgery (out of 19 patients treated with surgery) and 3 from other causes. The average duration of illness in those with fatal disease was around 6 years. Rubin et al. documented radiological progression in the cases who had repeat chest radiographs (Table 4). The rates of progression were proportional to the time between initial diagnosis and repeat x-ray, as shown in Figure 3 [20]. The development of fungal balls caused by *Aspergillus* spp. in histoplasmosis-related cavities has been documented in multiple cases [39].

**Table 4.** Frequency of Forms of Disease Progression on Chest X-ray in 87 Cases of Chronic Cavitary Pulmonary Histoplasmosis [20]

X-ray Progression	No. of Cases	%
New cavity formation	15	17
Spread to opposite lung	18	21
Spread to new area within the same lung	16	18
Cavity enlargement	11	13
Bronchopleural fistula with empyema	2	2
Destruction of lung by cavities	2	2

It has been noted that early cases may resolve spontaneously, leaving mild lung damage, but around 22% of patients progressed to cavitation. This would often result in progressive destruction of lung tissue leading to death unless treatment was given. However, 19% of patients whose early lesions resolved later developed new early lesions, with an average time gap of 6 years between episodes. Among those who did develop cavitary disease, thin-walled (1 mm) cavities were more likely to heal spontaneously and thick-walled ones (3–4 mm) required antifungal or surgical intervention more often [16].

### Treatment

A large review of 408 patients with CCPH treated with amphotericin B, surgery, or no treatment was performed by Parker et al. in 1970. In patients who did not have surgery, a negative prognostic factor was greater age at the time of diagnosis, and in those treated with amphotericin B, the extent of disease at the time of initiation of therapy was important. Death was less likely in patients treated with higher doses of amphotericin B, and death was less likely in those treated with amphotericin B than those who received no treatment [40].

Table 5 shows the outcomes in the case series by Goodwin et al. Goodwin et al. emphasized that their data could be not be used to compare antifungal and surgical therapy due to a variety of confounding factors. However, both persistent cavities and especially thick-walled cavities were less likely to heal without therapy [16]. Wheat et al. reported outcomes of cavitary disease in their study of CCPH following outbreaks of histoplasmosis. It should be noted that these outreak findings may not be extrapolated to all chronic cases. Spontaneous improvement and resolution were more common in patients with thinner cavities. The therapeutic agents used in these outbreaks were amphotericin B or ketoconazole. Outcomes are summarized in Table 6 [15].

**Table 5.** Treatment Results in 382 Lesions in 228 Patients With Chronic Cavitary Pulmonary Histoplasmosis [16]

		Conservative Rx		Amphotericin B Rx^a^		Surgical Rx	
Types of Lesions	No. of Lesions	No. of Lesions	% Healed	No. of Lesions	% Healed	No. of Lesions	% Healed
Early, no persistent cavity	156	139	99	6	100	11	100
Early, with persistent cavity	44	25	16	11	55	8	100
Late, no persistent cavity	41	36	100	0		5	100
Late, thin-walled cavity	52	27	63	12	92	13	100
Late, thick-walled cavity	89	53	21	15	63	21	95
Total lesions	382	280		44		58	

^a^Amphotericin B treatment: total dose range 1.7–2.5 g.

**Table 6.** Clinical Outcomes of Cases of Cavitary Histoplasmosis Following 2 Urban Outbreaks [15]

Outcome	No. of Cases	%
Spontaneous improvement	10	22.2
Spontaneous resolution	5	11.1
Improvement with treatment	15	33.3
Resolution with treatment	1	2.2
Persistence with treatment (>1 y)	2	4.4
Death from histoplasmosis	1	2.2
Death from other causes	2	6.7
Other^a^	8	17.8

^a^Response of some patients was still undetermined at the time of publication.

In the more recent review of cases by Kennedy et al., where cavitary disease affected far fewer patients and the predominant feature was nodules, most patients exhibited a good response to therapy. Of the 46 cases, 33 required therapy, of whom 24 patients showed good response to antifungal agents. Due to the nonrandomized nature of treatment and small sample size, no direct comparison of different antifungals could be made. In the 13 cases in which antifungal therapy was not used, there were 2 cases of progressive disease and 1 case of persistent x-ray abnormalities [19].

The 2007 update by the Infectious Diseases Society of America is the most recent recommendation of therapy for CCPH. It suggests itraconazole for at least 1 year, though it notes that some clinicians suggest continuation up to 2 years to avoid relapse [33].

### Research Needs

CPH can be a progressive disease resulting in enlarging cavities, loss of lung function, and mortality. Early diagnosis is key to prevention of deterioration, but the similarities to pulmonary TB may hinder recognition of CPH in low-resource settings where both TB and histoplasmosis are endemic. To raise awareness for the condition, an understanding of its incidence is crucial. Historical studies from the United States showed a rate of 3%–7% in patients with presumed TB [16, 22]. Contemporary studies that assess the incidence of CPH among patients presenting with presumed smear-negative pulmonary TB in low-resource settings are needed. Development of a point-of-care serological test would facilitate diagnosis in endemic areas. Finally, more data are needed on the natural course of CPH, including cavitary and nodular disease and response to treatment.

## CONCLUSIONS

CPH is a clinical entity that can remain undiagnosed and cause significant morbidity, and its prevalence is not known. Most information we have on the condition originates from historical case series. More contemporary data are needed in order to understand the prevalence of this disease and to be able to facilitate timely diagnosis and management.
